# Transferring ART research into education in Brazil

**DOI:** 10.1590/S1678-77572009000700017

**Published:** 2009

**Authors:** Maria Fidela de Lima NAVARRO, Karin Cristina da Silva MODENA, Maria Cristina Carvalho de Almendra FREITAS, Ticiane Cestari FAGUNDES

**Affiliations:** 1 DDS, PhD, Professor, Department of Dental Materials, Endodontics and Operative Dentistry, Bauru School of Dentistry, University of São Paulo, Bauru, SP, Brazil.; 2 DDS, PhD Student, Department of Operative Dentistry, Endodontics and Dental Materials, University of São Paulo, Bauru School of Dentistry, Bauru/SP, Brazil.; 3 DDS, MSc Student, Department of Operative Dentistry, Endodontics and Dental Materials, University of São Paulo, Bauru School of Dentistry, Bauru/SP, Brazil.; 4 DDS, MSc, PhD, Fellow, Department of Operative Dentistry, Endodontics and Dental Materials, University of São Paulo, Bauru School of Dentistry, Bauru/SP, Brazil.

**Keywords:** Dental caries, Atraumatic Restorative Treatment (ART), Dental restoration, Dental education, Oral health, Attitude

## Abstract

The aim of this study was to evaluate the teaching of the Atraumatic Restorative Treatment (ART) approach in Brazilian dental schools. Materials and Methods: A questionnaire on this subject was sent to Pediatric Dentistry, Operative Dentistry and Public Health Dentistry professors. The questions approached the followig subjects: the method used to teach ART, the time spent on its teaching, under which discipline it is taught, for how many years ART has been taught and its effect on the DMFT index. Results: A total of 70 out of 202 dental schools returned the questionnaire. The ART approach is taught in the majority of the Brazilian dental schools (96.3%), and in most of these schools it is taught both in theory and in clinical practice (62.9%). The majority (35.3%) of professors teach ART for 8 hours, and most often as part of the Pediatric Dentistry discipline (67.6%). It has been taught for the last 7 to 10 years in 34.3% of dental schools. Most professors did not observe a change in the DMFT index with this approach. There is a diversity in the teaching of ART in Brazil in terms of the number of hours spent, the teaching method (theory and practice), and the disciplines involved in its teaching. Conclusions: It is necessary to address the training of professors in the ART approach for the whole country. An educational model is proposed whereby a standard ART module features as part of other preventive and restorative caries care educational modules. This will facilitate and standardize the introduction and adoption of the ART approach in undergraduate education in Brazil.

## INTRODUCTION

Atraumatic Restorative Treatment (ART) takes a special place within the group of minimal intervention approaches for the management of dental caries^4^^,^^16^. This treatment approach was recognized and endorsed by the World Health Organization (WHO) for bringing restorative dental treatment to people who would not normally have access to dental care. The ART approach has become available through the combination of a better understanding of the dental caries process, permitting minimal cavity preparations and effective use of adhesive restorative materials^37^. The procedure involves removal of carious tooth tissue using hand instruments only, followed by restoration usually with a glass-ionomer cement^17^^,^^33^^,^^35^.

Critics to the ART approach argue that in spite of the positive results in research carried out into the use of the ART approach in clinical trials^9^^–^^11^^,^^14^^,^^16^^,^^20^^,^^36^^,^^38^, many dental institutions in Brazil do not include this approach in their *curricula*. There is, however, no reliable information about the teaching of the ART approach in Brazil with respect to the theory, its laboratory and clinical teaching.

Brazil is currently committed to the implementation of a Family Health Program (FHP) that aims to extend basic health care to the whole population. The FHP is changing from an emergency and restorative treatment model to one of disease prevention and health promotion for individuals as well as families and communities. This will make primary health care the foundation of the Brazilian healthcare system^21^. The main characteristics of the FHP are: a focus on the family, use of a multidisciplinary team, preventive activities, assessment of population needs and intersectoral action to promote health care^6^^,^^15^. In addition to the FHP, the Brazilian Government has also started the Programa Brasil Sorridente (Brazil Smiling Program) that has the objective of improving oral health care for the Brazilian population. It is the first time that the Federal Government has developed a national oral health policy with a well established program and not solely based on oral health care. With this program, in addition to basic dental care, the population has access to specialized treatments, such as management of oral cancer, endodontics, orthodontics and surgery^7^.

Thus, in Brazil, it is desirable to have, as soon as possible, dental practitioners who are competent theoretically and clinically in the ART approach, to enable them to implement the treatment required by the population under the responsability of each health team.

It was considered that a study regarding the teaching of ART in Brazil, covering all the regions of the country, would provide important information to health managers. This study might also enable the Brazilian authorities to find ways to facilitate the teaching and practice of ART in Brazilian dental institutions. Therefore, the aim of this study was to contact all dental schools in Brazil seeking information, through a questionnaire, from the professors from different disciplines regarding the teaching of ART at the undergraduate level, and to make recommendations based on the outcomes.

## MATERIAL AND METHODS

### Questionnaire Development

**FIGURE 1:**
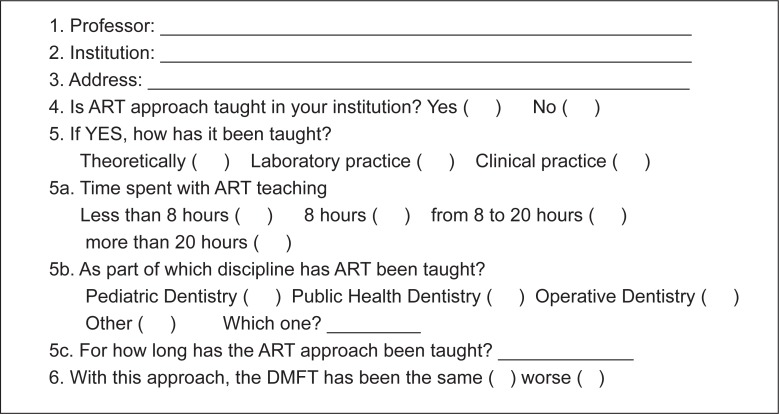
Questionnaire on teaching ART in dental institutions in Brazil

The authors prepared a short and straightforward questionnaire regarding the teaching of the ART approach, in such a way that professors could quickly and easily answer it. The questionnaire consisted of three sections: a) personal and institutional details; b) the method and the time used for teaching ART and the number of years the approach has been taught; and, c) the effects of ART on DMFT (Figure 1).

### Selection of the Study Population

According to the Ministry of Education (MEC), at the time of sending the questionnaires there were 202 dental schools in Brazil. Twenty were in the North, 34 in the Northeast, 15 in the Center-west, 101 in the Southeast and 32 in the South of the country. The target population comprised all dental professors working in Pediatric Dentistry, Public Oral Health, or Operative Dentistry departments from private, regional and district dental institutions in Brazil.

### Procedure for Obtaining the Names and Addresses of Dental Professors

The professors' e-mails were obtained from the websites of the universities and individual schools. When an institution did not have a website, or the names of professors were not readily available, the secretaries of the deans of these institutions were contacted by telephone to supply updated information about the professors' names and their electronic addresses. All addresses were entered into a computer database, using Microsoft excel software.

## RESULTS

A total of 70 of the 202 dental schools in Brazil answered the questionnaire, which represents an almost 35% response rate. The question: “Is the ART approach taught in your institution?” was answered by the majority as “yes” (96.3%); the remaining 3.7% answered “no”.

**FIGURE 2:**
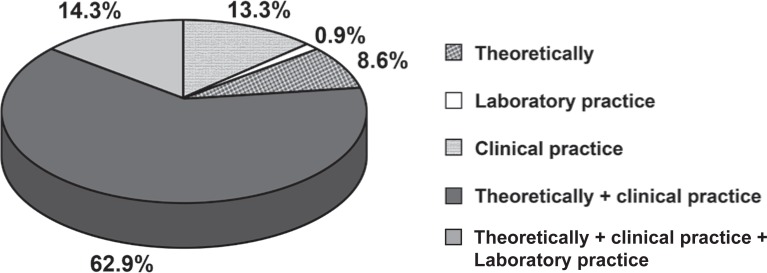
Answers given by professors from 70 dental schools in Brazil to the question “How is ART being taught?”

According to the respondents, ART is taught both in theory and in clinical practice in the majority of dental schools (62.9%). In 14.3% of the dental schools, ART is taught through a combination of theoretical teaching, laboratory and clinical practice. ART is taught only through theoretical teaching in 13.3% of dental schools, only clinical teaching in 8.6%, and only laboratory practice in 0.9% of schools (Figure 2).

**FIGURE 3:**
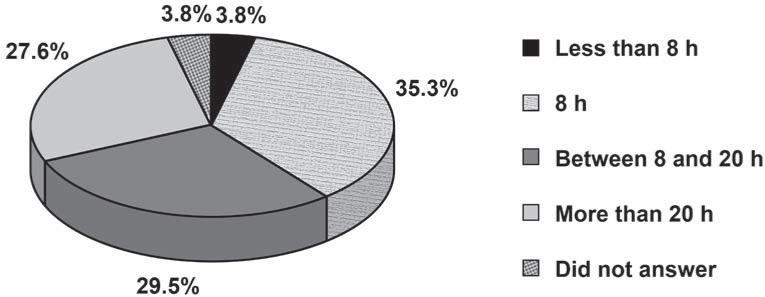
Percentage distribution of responses to the question “How much time is spent teaching ART”?

Regarding the time spent with ART teaching, the majority of dental schools answered “8 hours” (35.3%), followed by “from 8 to 20 hours” (29.5%), “more than 20 hours” (27.6%),” less than 8 hours” (3.8%), while 3.8% did not answer (Figure 3).

**FIGURE 4:**
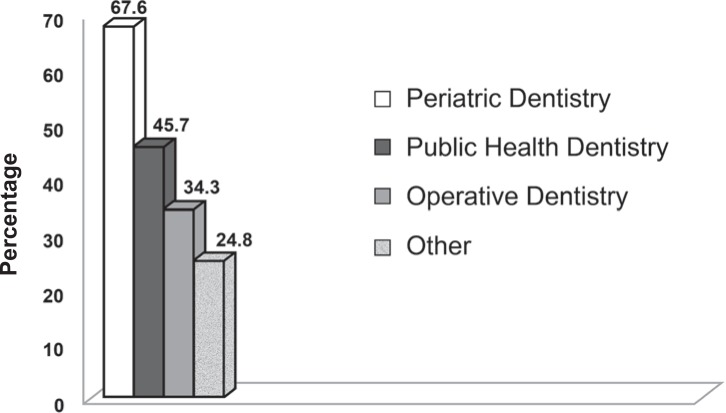
Distribution of the responses to the question “As part of which discipline has ART been taught?”

Figure 4 summarizes the responses to the question “As part of which discipline has ART been taught?”. The majority (67.6%) stated that ART is taught in “Pediatric Dentistry”, followed by “Public Health Dentistry” (45.7%), “Operative Dentistry (34.3%), and “Other disciplines” (24.8%).

**FIGURE 5:**
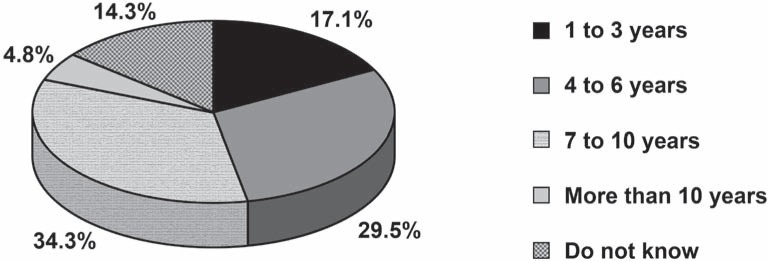
Percentage distribution of responses to the question “For how long has the ART approach been taught”?

With respect to the question “For how long has the ART approach been taught?”, the majority (34.3%) answered that this approach has been taught for “7 to 10 years”, while 29.5% answered “4 to 6 years”, 17.1% answered “1 to 3 years”, 4.8% stated that ART has been taught for “more than 10 years” and 14.3% did not know (Figure 5).

As regards the DMFT index, none of the respondents stated that the DMFT was worse because of the ART approach. The majority (66.7%) answered that the index had remained the same, and 33.3% did not know.

## DISCUSSION

Since its introduction, the ART approach has become a well established caries management option (preventive and restorative), even though it might have some limitations under certain situations. ART is based on a preventive philosophy which includes early interception of the carious process by using different types of fluorides and, when necessary, minimally invasive intervention to conserve sound tooth tissue^27^. Thus, ART must not be used in isolation but should be included with preventive programs and health education to be effective by controlling the etiologic factors of caries. The educational activities and preventive procedures include diet counseling, oral hygiene instruction, plaque removal, and use of remineralizing agents^40^.

The ART approach has been incorporated in undergraduate curricula in a number of dental schools around the world. examples are, Thamarasset Dental School in Thailand and Muhimbili Dental School in Tanzania^23^^,^^24^. The approach is taught in dental schools with the aim of promoting public health to those who do not normally have access to oral health treatment^12^.

In this context, many countries have shown dissatisfaction regarding the insignificant contribution of preventive and restorative care to the oral health of their population. Approximately two-thirds of the world population do not have any professional oral care^32^. For example, twenty seven million Brazilians, almost 15% of the total population, have never received any dental treatment, according to a demographic survey performed in 1998^22^. Since then the number of Brazilians who have access to oral health programs of prevention and treatment of oral diseases has increased. For this sector of the population, the Family Health Program (FHP) and the “Brazil Smiling Program” that the Brazilian federal government has established, will take trained medical and dental practitioners to rural and suburban areas where the population does not have access to health treatment. The ART approach was originally developed for this sector of the population so it is necessary to know if dental schools are teaching this approach to their students.

There are many studies that have used questionnaires to evaluate the curricular structure, teaching philosophies, knowledge, the skills of teaching, the status and factors associated with organizational innovation in dental schools^1^^,^^25^^,^^29^. In the present study, data was collected from nearly 35% of all dental schools in Brazil, which represents 70 schools. Although in our study we used a short questionnaire, with the intention of improving the response rate, other similar studies have had a response rate ranging from 70.5% to 100%^1^^,^^25^^,^^29^. This difference in response rates may have occurred because Brazilian people are resistant to answering questionnaires for evaluation; for example, some studies have responses as low as 8.4%^34^, 35%^13^ and 39.5%^3^. Another possible reason is that some of the non-respondent dental schools might not as yet have incorporated ART within their curricula and were reluctant to report on this.

The high percentage (96.3%) of the responding dental schools that teach ART to their students reveal the importance that their professors attach to ART. The majority of the professors (62.9%) teach the ART approach only theoretically and clinically, however laboratory practice is important to teach some of the finer details that the approach requires^27^, such as proper cavity cleaning (preparation) and glass ionomer cement manipulation.

Most dental schools claim that they spend between 8 and 20 hours on the teaching of ART. We believe that a minimum of 8 hours for theory and 8 hours for laboratory practice are sufficient to develop good skills with the approach. However, more time should be spent for developing clinical skills since the student can encounter many different situations and difficulties^26^ when applying the ART approach, such as different occlusal access^31^, consistency and depth of the dentin lesion^5^.

The ART approach was developed in Tanzania in the mid-1980's^16^^,^^18^. However, it was only in 1994 that the WHO recognized it as a revolutionary technique for caries lesion treatment^37^. The Brazilian dental schools delayed some years before including ART on their undergraduate curricula but even so the majority of dental schools have been teaching the ART approach for between 4 to 10 years. There remains a diversity in terms of hours spent, kind of teaching (theory and practice) and disciplines involved. Ideally, if the school really accepted the ART approach, all the disciplines cited (Pediatric Dentistry, Public Health Dentistry and Operative Dentistry) should teach this approach. This fact points out that it is necessary to address a training of professors covering the whole country.

In 1993, 1996 and 2003, the DMFT (at 12 years of age) reported for Brazil was 4.90, 3.06 and 2.78, respectively, according to the Ministry of Health of Brazil^8^. This shows a clear decrease of the DMFT throughout the years that is associated with many factors, including the use of fluoride in the drinking water, the use of fluoride toothpastes, and the implementation of new government programs focused on oral health. The majority of the professors did not note differences in the DMFT after the introduction of the ART approach because it is part of health programs. Considering the FHP and “Brazil Smiling Program”, it would be very important to motivate professors from target groups, as those from Pediatric Dentistry, Operative Dentistry and Public Health Dentistry, and practitioners already working in these programs to participate in a training covering the whole country using tools such as e-learning with classes addressed by expert professors and books or printed material regarding the ART, aiming to get the best of the ART approach^17^^,^^18^^,^^30^. In these classes the survival rate of ART restorations in different clinical trials should be stressed^2^^,^^19^^,^^39^.

Based on the current knowledge on the state of the ART approach and on the experiences of dental schools that have introduced ART in the curriculum, an educational model presenting the ART features as part of restorative and preventive caries care modules should be established to facilitate and standardize the introduction and adoption of the ART approach in the undergraduate education in Brazil.

## CONCLUSIONS

There is a diversity in the ART teaching in Brazil in terms of hours spent, kind of teaching (theory and practice), and disciplines involved. It is necessary to address a training of professors covering the whole country. An educational model presenting the ART features as part of restorative caries care modules should be established to facilitate and standardize the introduction and adoption of the ART approach in the undergraduate education in Brazil.
